# Establishing patient-derived organoids from human endometrial cancer and normal endometrium

**DOI:** 10.3389/fendo.2023.1059228

**Published:** 2023-04-14

**Authors:** Arielle Katcher, Brian Yueh, Kadir Ozler, Aaron Nizam, Ariel Kredentser, Charlie Chung, Marina Frimer, Gary L. Goldberg, Semir Beyaz

**Affiliations:** ^1^ Cold Spring Harbor Laboratory, Cold Spring Harbor, NY, United States; ^2^ Department of Obstetrics and Gynecology, Division of Gynecologic Oncology, Long Island Jewish Medical Center, Zucker School of Medicine at Hofstra/Northwell, Northwell Health, New Hyde Park, NY, United States; ^3^ Institute for Molecular Medicine, Feinstein Institutes for Medical Research, Manhasset, NY, United States

**Keywords:** organoid, endometrial cancer, endometrium, patient derived, organoid culture method

## Abstract

Endometrial cancer is the most common gynecologic malignancy in the United States and is one of the few malignancies that had an increasing incidence and mortality rate over the last 10 years. Current research models fail to recapitulate actual characteristics of the tumor that are necessary for the proper understanding and treatment of this heterogenous disease. Patient-derived organoids provide a durable and versatile culture system that can capture patient-specific characteristics such as the mutational profile and response to therapy of the primary tumor. Here we describe the methods for establishing, expansion and banking of endometrial cancer organoids to develop a living biobank. Samples of both endometrial tumor tissue and matched normal endometrium were collected from 10 patients. The tissue was digested into single cells and then cultured in optimized media to establish matched patient endometrial cancer and normal endometrial tissue organoids. Organoids were created from all major endometrial cancer histologic subtypes. These organoids are passaged long term, banked and can be utilized for downstream histological and genomic characterization as well as functional assays such as assessing the response to therapeutic drugs.

## 1 Introduction

The human endometrium is the dynamic hormone responsive cellular lining of the uterus. Several disease processes originate in the endometrium including malignancy. Endometrial cancer is the most common gynecologic cancer and the fourth most common cancer among women in the United States (1, 2). Incidence of endometrial cancer and disease-associated mortality has increased worldwide and rates are projected to continue to rise in coming years (1). The incidence of disease peaks in women aged 60 to 70 years old (3).

Obesity is one of the leading risk factors for endometrial cancer and has one of the strongest associations with obesity amongst other cancer types including colorectal, breast, and ovarian cancer (2–4). The obesity epidemic drives the increasing rate of endometrial cancer seen in the general population. In the United States, 57% of all endometrial cancers may be attributable to obesity (4).

Historically, endometrial cancer has been classified into two subtypes. Type I endometrial cancers make up most of the endometrial cancers (80%), show endometrioid histology and are associated with a more favorable clinical prognosis. Type II cancers are less common (10-20%), and are comprised of high risk, high grade histologies and they are associated with a worse prognosis. Although type II endometrial cancers comprise a smaller percentage of all endometrial cancers, they represent up to 40% of the morbidity and mortality and they disproportionately affect minority African American women (5, 6).

With the rising rates of endometrial cancer, there remains an unmet need for reliable biologic models to study the process of cancer formation, tumor metastasis, and disease progression and recurrence. There is limited data available about the molecular and cellular basis for the establishment of endometrial cancers especially in the rarer and more aggressive subtypes. This is partly attributable to a lack of high-fidelity models that can accurately mimic the *in vivo* properties of human endometrial cancer and normal endometrium. Immortalized 2D cell lines such as Ishikawa cells have been used for *in vivo* study of endometrial cancer (7). One advantage of these lines is that they can be cultured for long periods of time and are easy to store. However, these cell lines are subject to transformation in culture and lose characteristics of the primary tumor. 2D cell culture systems also lack the important 3D structure that is represented in primary endometrial tissue where structure greatly influences function of the glandular cells as their microenvironment and orientation determine function (8, 9). Mouse models have been used for the study of endometrium, however, there are significant biologic differences between mouse and human endometrium. Namely, human endometrium is shed cyclically due to the hormonal milieu while mouse endometrium is only shed in settings of pregnancy or trauma (10–12).

3D organoids can recapitulate the microenvironment and cellular milieu more accurately than 2D models allowing for a more similar representation of the endometrium *in vivo* (13, 14). Additionally, the establishment of patient derived organoids allows the cells to be maintained in culture and therefore the limitation of tissue sample is overcome by a renewable cellular resource (15). Organoids have been shown to maintain their phenotype and genotype throughout passaging, making them a reliable model that maintains its biological integrity while being grown in culture over many passages (16). The application of organoid technology is vast and extends beyond the study of cancer. In the realm of cancer biology, organoids can be used for *in vitro* testing of new therapeutics that can be tailored towards specific histologic and molecular subtypes of cancer (17). Here we describe an optimized approach to establishing, expanding and banking endometrial organoids derived from patient samples of normal endometrium and endometrial tumors.

## 2 Materials and equipment

### 2.1 Reagents

1. Advanced DMEM/F12 (Life Technologies, Catalog # 12634028)
2. Glutamax (Life Technologies, Catalog # 35050061)
3. N2 Supplement (Life Technologies, Catalog # 17502048)
4. B27 Supplement minus Vitamin A (Life Technologies, Catalog # 12587010)
5. Chemically Defined Lipid Concentrate (Life Technologies, Catalog # 11905031)
6. N-Acetyl-L-cysteine (Sigma Aldrich, Catalog # A9165-5G)
7. ALK-4, -5, -7 inhibitor, A83-01 (Sigma Aldrich, Catalog # SML0788)
8. Nicotinamide (Sigma Aldrich, Catalog # N0636)
9. B-Estradiol-Water Soluble (Sigma Aldrich, Catalog # E4389)
10. SB202190 (p38i) (Sigma Aldrich, Catalog # S7067)
11. HEPES (Sigma Aldrich, Catalog # S7067)
12. Collagenase from Clostridium histolyticum (Sigma Aldrich, Catalog # C9407)
13. Recombinant Human IGF-1 (Peprotech, Catalog # 100-11)
14. Recombinant Human EGF (Peprotech, Catalog # AF-100-15)
15. Recombinant Human Noggin (Peprotech, Catalog # 120-10c)
16. Recombinant Human FGF-10 (Peprotech, Catalog # 100-26)
17. Recombinant Human FGFb (Peprotech, Catalog # 100-18B)
18. Recombinant Human HGF (Peprotech, Catalog # 100-39)
19. Y-27632 dihydrochloride (Tocris, Catalog # 1254)
20. Primocin (*In vivo*gen, Catalog # ant-pm-1)
21. Cell Recovery Solution (Corning, Catalog # 354253)
22. Recovery™ Cell Culture Freezing Medium (Thermo Fisher Scientific, Catalog # 12648010)
23. TrypLE™ Express Enzyme (1x), no phenol red (Thermo Fisher Scientific, Catalog # 12604103)
24. Insulin-Transferrin-Selenium (ITS-G) (100X) (Thermo Fisher Scientific, Catalog # 41400045)
25. Matrigel^®^ Matrix (Corning, Catalog # 356234)
26. RPMI-1640 Medium (Sigma Aldrich, Catalog # R73-88)
27. Phosphate Buffered Saline (PBS)
28. Paraformaldehyde (PFA) (Electron Microscopy Sciences, Catalog # 15714)
29. Agarose gel (BioExcell, Catalog # A-1705)

### 2.2 Equipment

1. Falcon 15cm Dish (Corning, Catalog # 430599)
2. Falcon 15mL Tube (Corning, Catalog # 352097)
3. Falcon 50mL Tube (Corning, Catalog # 352098)
4. Cell Lifter (Corning, Catalog # 3008)
5. 6 Well Plate (Corning, Catalog # 353046)
6. 12 Well Plate (Corning, Catalog # 353043)
7. 24 Well Plate (Corning, Catalog # 353047)
8. Eppendorf 1.5mL Tube (Eppendorf, Catalog # 22364116)
9. CRYOVIAL^®^ Internal Thread with Silicone Washer Seal (Simport, Catalog # T311-1)
10. CO_2_ Incubator
11. Light Microscope
12. Water Bath
13. Centrifuge
14. Pipette
15. Forceps
16. Scissors

A83-01: Dissolve 10mg in 474.47uL DMSO to make a 50mM solution.

B-estradiol-Water Soluble: Dissolve 100mg in 4mL DH20 to make a 25mg/mL solution.

Collagenase: Dissolve 100mg in 10mL RPMI to make a 10mg/mL solution.

Matrigel^®^: Thaw the original bottle overnight at 4°C on ice. Make 1ml aliquots.

N-Acetyl-L-cysteine: Dissolve 1g in 10mL DH20 to make a 612.7mM solution.

Nicotinamide: Dissolve 122.12mg in 1mL DH20 to make a 1M solution.

Recombinant Human EGF: Dissolve 500ug in 500uL PBS + 0.1% BSA to make a 1mg/mL solution.

Recombinant Human FGF-10: Dissolve 50ug in 50uL PBS + 0.1% BSA to make a 1mg/mL solution.

Recombinant Human FGFb: Dissolve 10ug in 100uL 5mM Tris with pH of 7.6 to make a 0.1mg/mL solution. Then dilute with 100uL PBS + 0.1% BSA to make a 0.05mg/mL solution

Recombinant Human HGF: Dissolve 100ug in 200uL of DH20 to make a 0.5mg/mL solution. Let sit at RT for one hour. Dilute with 200uL PBS +0.1% BSA to make a 0.25mg/mL solution.

Recombinant Human IGF-I: Dissolve 100ug in 100uL PBS + 0.1% BSA to make a 1mg/mL solution.

Recombinant Human Noggin: Dissolve 250ug in 250uL PBS + 0.1% BSA to make a 1mg/mL solution.

SB202190: Dissolve 5mg in 754.512uL DMSO to make a 20mM solution.

Y-27632: Dissolve 10mg in 299.607uL of DH20 to make a 100mM solution.

ACK lysis buffer: Home-made. Filter through a 0.2um mesh prior to use.

R-Spondin conditioned media: Home-made (Kuo et al.) Filter through a 0.2um mesh prior to use.

Creation of Normal Endometrium and Endometrial Tumor Media

Normal Endometrium Medium:

Medium should be stored at 4°C for a maximum of 10 days. Combine 1mL B-27 Supplement, 500uL N2 Supplement, 500uL ITS-G, 102uL N-Acetyl-L-Cysteine, 100uL Primocin, 100uL Nicotinamide, 25uL SB202190, 5uL Recombinant Human Noggin, 5uL Y-27632 dihydrochloride, 2.5uL Recombinant Human EGF, 2uL Recombinant Human FGFb, 1uL Recombinant Human FGF-10, 0.5uL A83-01, 0.5uL B-estradiol, 10mL R-Spondin Conditioned Media, 37.5mL ADMEM with 1% Penicillin/Streptomycin, 10mM HEPES, and 2mM Glutamax.

Tumor Endometrium Medium:

Medium should be stored at 4o C for a maximum of 10 days. Combine 1mL B-27 Supplement, 500uL N2 Supplement, 500uL Lipid Concentrate, 102uL N-Acetyl-L-Cysteine, 250uL Nicotinamide, 100uL Primocin, 5uL B-estradiol, 5uL Recombinant Human Noggin, 5uL Y-27632 dihydrochloride, 4uL Recombinant Human HGF, 2.5uL Recombinant Human EGF, 2uL Recombinant Human IGF-1, 0.25uL SB202190, 0.25uL A83-01, 10mL R-Spondin Conditioned Media, 37.5 mL ADMEM with 1% Penicillin/Streptomycin, 10mM HEPES, and 2mM Glutamax.

## 3 Methods

### 3.1 Human specimens

Fresh tumor specimen was obtained from 10 patients undergoing hysterectomy for endometrial cancer. Institutional Review Board approval was obtained (study IRB #18-0897) and all patients provided informed consent prior to specimen collection. All specimens were delivered from Long Island Jewish Medical Center to Cold Spring Harbor Laboratory on the day of collection or the following day. Biopsies were obtained from surgical hysterectomy specimens measuring at least 2 x 2 x 2mm in size. Collected tissue was transported in a solution of RPMI-1640 Medium (Sigma Aldrich, Catalog # R73-88), 1% Penicillin/Streptomycin, 10mM HEPES (Sigma Aldrich, Catalog # S7067), and 2mM Glutamax (Life Technologies, Catalog # 35050061). If tissue was unable to be delivered on the day of the surgery, it was stored overnight at 4°C.

### 3.2 Tissue digestion and organoid culture

#### 3.2.1 Establishment of endometrial organoids from surgical samples, time: 3-4 hours

**Figure 1:**
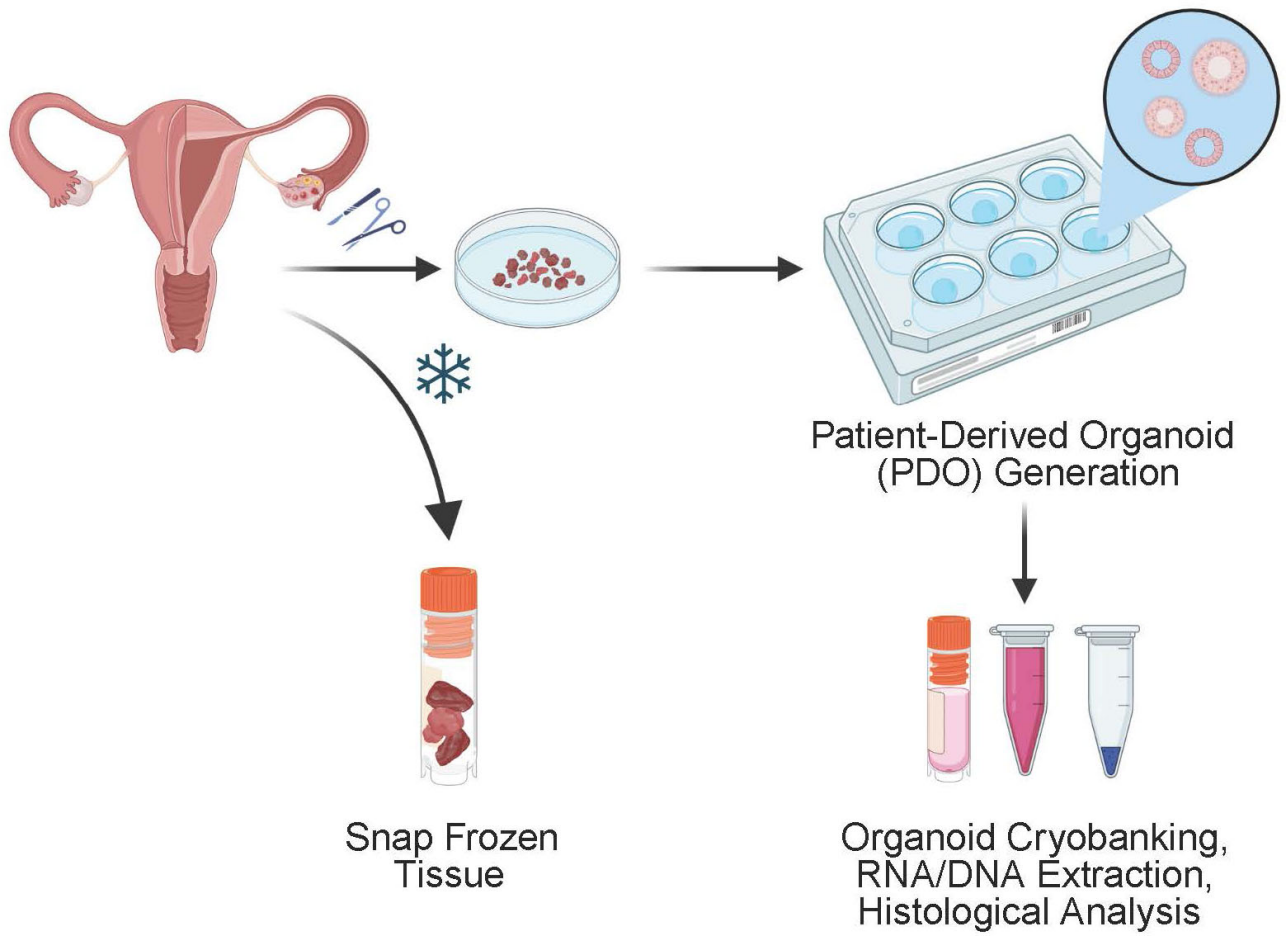
Schematic of the generation of endometrial organoids derived from patient surgical specimens. Subsequent culturing is performed to create patient derived organoids which can be further analyzed for DNA and RNA extraction and histological analysis. Remaining specimen is frozen for storage and can be thawed and used for continued tissue culture.

Benign endometrial tissue and endometrial tumor tissue is received and processed separately by first washing twice with PBS. Tissue is then minced in a petri dish with shears into 1mm^3^ fragments or smaller (**Figure 1**). The fragments are transferred to a 15mL Falcon tube with 5mL of collagenase solution (1mg/mL) and 10uM Y-27632 dihydrochloride (Tocris, Catalog # 1254) in RPMI-1640 Medium (Sigma Aldrich, Catalog # R73-88). Endometrial glands are incubated in the solution at 37°C for 90-150 minutes and intermittently mixed every 15 minutes. Digestion is evaluated with light microscopy for dissociated fragments. The supernatant is transferred to a separate 15 mL Falcon tube and centrifuged at 300 x g for 5 minutes at room temperature. Next the supernatant is removed and 1mL of TrypLE™ Express Enzyme (1X, no phenol red, Thermo Fisher Scientific, Catalog # 12604103) and 10uM Y-27632 dihydrochloride is added per 1cm^3^ of remaining tissue. The mixture is incubated at 37° C for and additional 10-20 minutes and evaluated for digestion with light microscopy every 10 minutes for dissociation to single cells. The mixture is pipetted every 5-10 minutes to ensure uniform digestion. Once the presence of at least 80% single cells are present in the mixture, quench the reaction by adding ADMEM/F12 (Life Technologies, Catalog # 12634028). The mixture is centrifuged at 300 x g for 5 minutes at room temperature. The pellet is assessed for the presence of red blood cells. If present, the pellet is resuspended in 1-2 mL of ACK Lysis Buffer for 2 minutes at room temperature and then, centrifuged at 300 x g for 5 minutes at room temperature. Next the pellet is resuspended evenly in a 70:30 mixture of Matrigel^®^ Matrix (Corning, Catalog # 356234): Organoid media and placed on ice to prevent polymerization of the mixture prior to plating. The mixture is plated into 50uL domes on a prewarmed 6 well plate (Corning, Catalog # 353046). The plate is placed in a CO_2_ incubator (37° C, 5% CO_2_) for 10 minutes to allow the Matrigel to polymerize and solidify following which 3mL of normal endometrial organoid media or endometrial tumor organoid media is added to the normal and tumor endometrial organoid wells respectively.

#### 3.2.2 Passaging endometrial organoids, time: 1.5-2 hours

First, the organoid media is removed from the wells. The Matrigel domes are collected and mixed using a pipette with 1mL of Cell Recovery Solution (Corning, Catalog # 354253) per well and the mixture is allowed to rest on ice for 30-60 minutes during which the mixture is resuspend with a P1000 pipette every 15 minutes. Once the Matrigel is completely dissolved the mixture is centrifuged at 500 x g for 5 minutes at 4° C. The supernatant is removed and 400uL TrypLE and Y-27632 dihydrochloride is added for every well collected. The mixture is incubated at 37° C for 10-20 minutes and evaluated for digestion with light microscopy for dissociation into single cells. Once the presence of at least 80% single cells are present in the mixture, quench the reaction by adding ADMEM/F12 (Life Technologies, Catalog # 12634028). The mixture is centrifuged at 300 x g for 5 minutes at room temperature. The pellet is resuspended evenly in a 70:30 mixture of Matrigel^®^ Matrix (Corning, Catalog # 356234): Organoid media and placed on ice to prevent polymerization of the mixture prior to plating. The mixture is plated into 50uL domes on a prewarmed 6 well plate (Corning, Catalog # 353046). The plate is placed in a CO_2_ incubator (37° C, 5% CO_2_) for 10 minutes to allow the Matrigel to polymerize and solidify following which 3mL of normal endometrial organoid media or endometrial tumor organoid media is added to the normal and tumor endometrial organoid wells respectively.

#### 3.2.3 Freezing organoids, time: 1.5 hours

The organoid media is removed from the wells and the Matrigel domes are collected and mixed with Cell Recovery Solution 1mL per well (Corning, Catalog #354253) and left to sit on ice for 30-60 minutes while intermittently mixing with a P1000 pipette every 15 minutes. The mixture is centrifuged at 500 x g for 5 minutes at 4° C and the supernatant is removed. The pellet is resuspended in Recovery™ Cell Culture Freezing Medium (Thermo Fisher Scientific, Catalog # 12648010) and 500-1000uL of cells mixed in solution are transferred per 1.2mL Cryovial. Cryovials are stored into an established freezing container at -80° C for 24 hours and then the Cryovials are transferred to Liquid Nitrogen within the next 24 hours of storage.

#### 3.2.4 Thawing frozen organoids, time: 30-60 minutes

Cryovials are removed from liquid nitrogen storage and warmed in a 37° C water bath until only a small amount of ice remains. 1mL of organoid culture medium is added dropwise to the cryovial and the contents are transferred to a 1.5mL Eppendorf tube. The mixture is centrifuged at 300 x g for 5 minutes at room temperature. The pellet is resuspended in a 70:30 ratio of Matrigel : Organoid Media mixture and plated into 50uL domes on a pre-warmed six well plate. The plate is incubated in a CO_2_ incubator (37° C, 5% CO_2_) for 10 minutes to allow the Matrigel to solidify and 3mL of normal endometrial organoid media or endometrial tumor organoid media is added to the normal and tumor endometrial organoid wells respectively.

### 3.3 RNA isolation

RNA extraction was performed on organoids derived from both benign endometrial tissue and endometrial tumor tissue. Culture media is aspirated and Matrigel domes containing organoids are resuspended with 3X volume of Cell Recovery Solution (Corning, Catalog #354253) and incubated on ice for 1 hour. The resuspension is centrifuged at 500 x g for 5 minutes at 4° C. The supernatant is removed and the pellet is resuspended in 400uL of TRIzol™ Reagent (Fisher Scientific #15-596-018<tel:15-596-018>) and mechanically agitated to promote cellular lysis.

Total RNA is isolated using the Zymo Direct-zol RNA Microprep Kit (Zymo #R2062) according to the manufacturer’s instructions. Isolated RNA is used directly or stored in -80° C.

### 3.4 Preparation of organoids for imaging

#### 3.4.1 Preparing organoids for histology, time: 120-150 minutes

Culture media is aspirated from selected cell culture wells and Matrigel domes containing organoids are resuspended with 3X volume of Cell Recovery Solution (Corning, Catalog #354253) and incubated on ice for 1 hour. The resuspension is centrifuged at 500 x g for 5 minutes at 4° C. The supernatant is removed and the pellet is resuspended with 500uL of 4% paraformaldehyde (PFA) which is left at room temperature for 30 minutes to fix the cells. 300uL of PBS and 200uL of ADMEM/F12 (Life Technologies, Catalog # 12634028) is then added to the mixture. The mixture if centrifuged at 300 x g for 5 minutes at 4° C and the supernatant is discarded. 2% agarose gel is heated until uniformly liquified and 50uL is added to the organoid pellet and lightly mixed to suspend the organoids. The mixture is left at room temperature for one minute to solidify following which 100uL of PBS is added on top of the agarose to prevent drying. The mixture can then be processed for cut into sections and stained or stored at 4° C until ready for use.

## 4 Results

**Figure 2:**
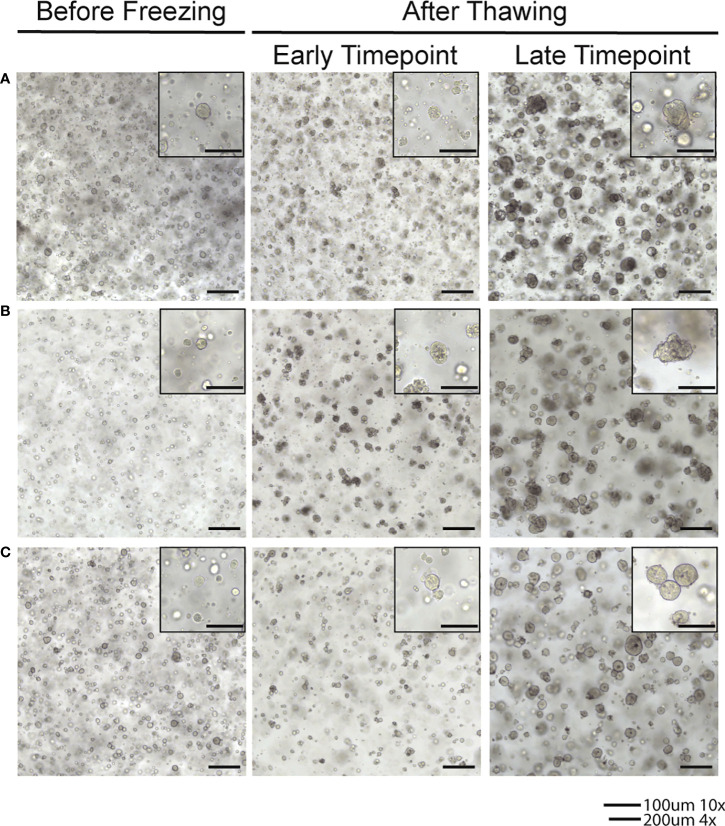
Brightfield images of organoids in culture after thawing from frozen samples. **(A)** Line frozen after 8 passages established in culture. **(B)** Line frozen after 9 passages established in culture. **(C)** Line frozen after 10 passages established in culture.

Endometrial tumor samples and matched normal endometrial tissue samples were received from consented patients who underwent surgical resection of endometrial cancer. The tissue was used to establish and culture organoids. Organoids were created from a diverse patient population with all racial and ethnic groups represented. Additionally, all major endometrial cancer histologic subtypes were represented in our population including endometrioid adenocarcinoma (Grades 1, 2, and 3), serous, clear cell, undifferentiated, dedifferentiated, and carcinosarcoma. Our culture medium was optimized to support the growth of both normal endometrial and endometrial tumor organoids. Media was changed every three to four days after plating to ensure adequate amounts of components for optimal growth and health of the organoids. After freezing organoids, we have demonstrated the ability to thaw and reestablish cultures with maintained phenotypes of the original organoid line (**Figure 2**).

**Figure 3:**
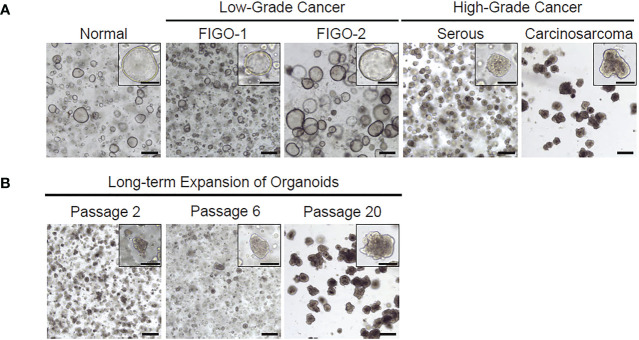
Representative images of patient-derived organoids from normal endometrium, low-grade cancer or high-grade cancer. **(A)** Both low and high grade histologic subtypes are able to be cultured and maintained through several passages. **(B)** Organoids can survive in culture over several passages.

**Figure 4:**
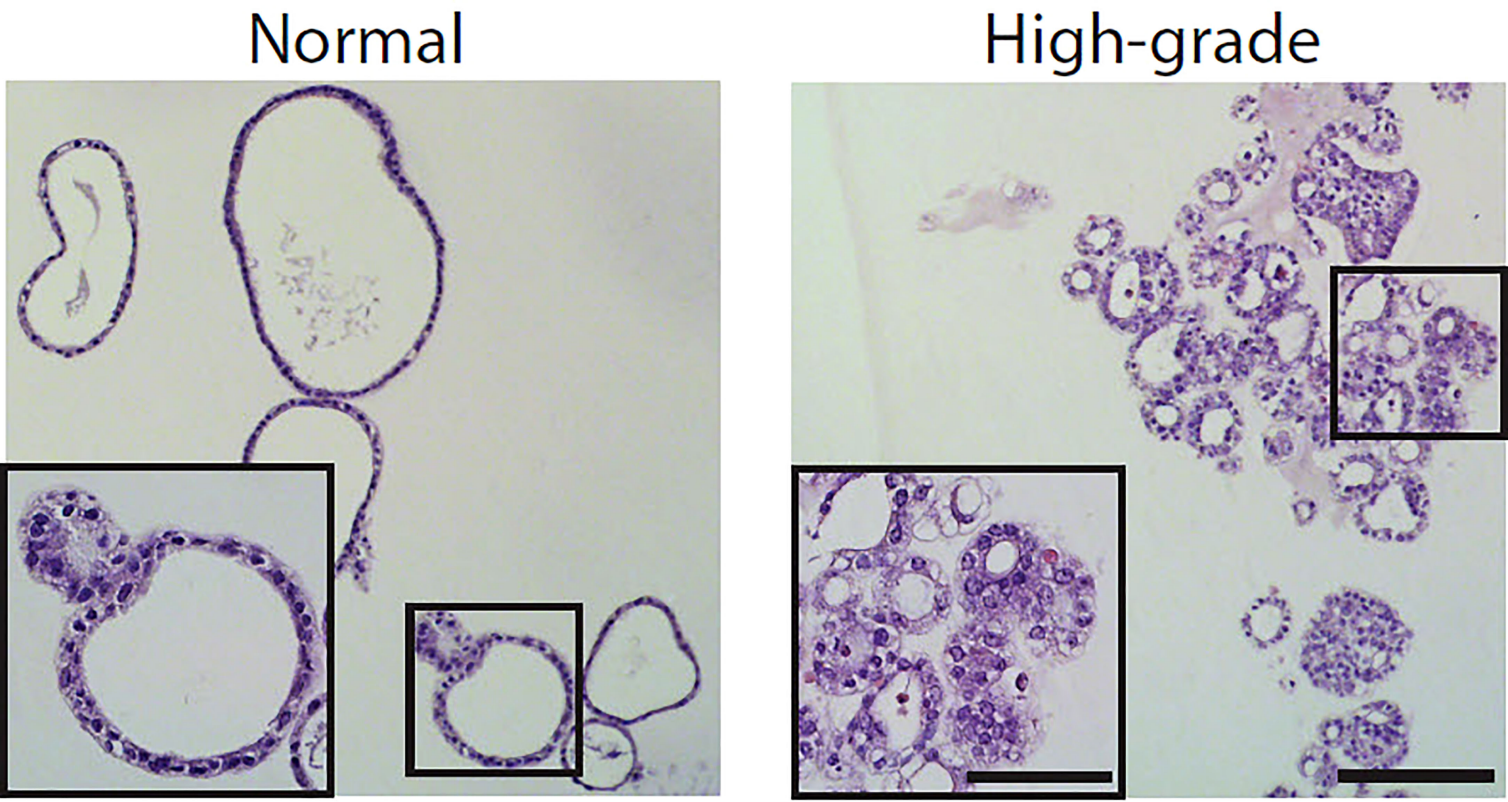
H&E staining of both normal endometrial derived organoids and high grade endometrial cancer derived organoids.

While in culture, endometrial organoids formed the characteristic spheroid structures on average three to four days after plating. Normal endometrium and low-grade tumors established larger hollow phenotype while high grade endometrial tumor organoids appeared smaller and more solid (**Figures 3**, **4**). This phenotype was noted to be maintained over multiple passages and also recapitulated after frozen organoids were thawed and cultured. Cultures of both benign endometrial tissue and endometrial tumor tissue were maintained for different time intervals depending on planned usage. The longest organoid culture to date still in maintenance at three years was derived from a high-grade endometrial cancer specimen. Organoids were split and passaged on average after different intervals depending on the appearance of the organoids to maintain the culture and ensure the health of the organoids as well as the type of organoid. On average, endometrial cancer organoids were passaged every 15 days and normal endometrial organoids were passaged every 12 days.

## 5 Discussion

We describe a protocol for establishing both normal endometrium and endometrial tumor organoids from patient derived tissue samples. The establishment of organoids is a critical step in pre-clinical testing as reliable biologic models are a mainstay of the study of tumor biology and the development of cancer therapeutics. Within the realm of endometrial cancer therapy, a lack of effective pre-clinical models that accurately mimic tumor biology has presented a unique unmet need. The development of patient derived organoids has changed the landscape for the study of endometrial cancer. Several studies have described the development and maintenance of patient derived endometrial organoids derived from both normal and tumor tissues (13, 16). Our own work and establishment of a biobank aims to further advance the field of organoid technology by increasing the diversity of patient specimens in patient race and ethnicity and in histologic subtype of the primary cancer tissue. Several clinical studies have shown that patient race and histologic subtype of cancer are among two of the most important qualities that effect clinical outcome and prognosis in women with endometrial cancer (18, 19). Therefore, establishing organoids created from a wide range of patients can lead to the study of specific factors that may affect outcomes in these predefined groups. Our organoids are amenable to drug testing as well as further experimentation without being limited by the quantity of the tissue sample provided from the initial specimen. Additionally, our organoid model can be applied to other endometrial pathology to study disease processes outside of cancer including endometriosis and infertility. The study of endometrial cancer through the use of organoids presents a unique and dynamic tool in trying to elucidate the complex disease processes of endometrial cancer. In the future we hope to apply this understanding to the discovery of novel treatments which can lead to meaningful clinical responses.

## Data availability statement

The raw data supporting the conclusions of this article will be made available by the authors, without undue reservation.

## Ethics statement

The studies involving human participants were reviewed and approved by Northwell Health Institutional Review Board. The patients/participants provided their written informed consent to participate in this study.

## Author contributions

AKa wrote the first and final drafts of the manuscript. AKa and AN wrote sections of the manuscript. SB, MF, and GG contributed to conception and design of the study. All authors contributed to the article and approved the submitted version. 
